# An overview of the feasibility of nanomedicine in pancreatic cancer theranostics

**DOI:** 10.37349/etat.2025.1002326

**Published:** 2025-06-18

**Authors:** Kyriakos Kokkinogoulis, Aristomenis Kollas, David Simeonidis, Pavlos Papakostas, Kalliopi Platoni, Efstathios P. Efstathopoulos, Mersini Makropoulou

**Affiliations:** IRCCS Istituto Romagnolo per lo Studio dei Tumori (IRST) “Dino Amadori”, Italy; ^1^Department of Physics, Faculty of Applied Mathematical and Physical Sciences, National Technical University of Athens, 15772 Athens, Greece; ^2^2^nd^ Oncology Clinic, Metropolitan General, 15562 Athens, Greece; ^3^2^nd^ Department of Radiology, Medical Physics Unit, Medical School, National and Kapodistrian University of Athens, 12462 Athens, Greece

**Keywords:** Nanomedicine, pancreatic cancer, personalized medicine, photothermal effect, photodynamic therapy, plasmonic photothermal therapy

## Abstract

Pancreatic ductal adenocarcinoma (PDAC) is among the top causes of cancer-induced mortality, frequently diagnosed too late to be treated effectively, due to the poor prognosis and the limited successful therapeutic options. Apart from the conventional treatments, new multimodal therapies have emerged utilizing different scientific fields for the improvement of the survival and quality of patients’ lives. The advancement of nanotechnology leads the way to more personalized medicine and the use of targeted theranostics carriers for deep-seated cancers such as PDAC. New nanotechnology innovations such as specialized photo-sensitizing drug nanocarriers, can effectively improve photodynamic therapy (PDT) of PDAC and enhance phototherapy’s action through surface plasmon resonance phenomenon, as another recently re-emerged non- or minimally invasive possible treatment of such diseases. Despite the scientific advancements, significant hurdles remain and many parameters need to be examined. However, the novel application of nano-biophotonic techniques and the convergence of different science fields offer promise for the treatment of difficult-to-treat diseases, like PDAC.

## Introduction

Pancreatic cancer (PaCa) is one of the foremost contributors to cancer-associated deaths, despite the significant advances in cancer research worldwide. According to the World Cancer Research Fund International (WCRFI) up-to-date PaCa statistics, PaCa ranked as the 12th most frequent cancer globally in 2020. It was the 12th leading cancer in men and the 11th most diagnosed cancer in women [1]. To give some numerical data, over 495,000 new PaCa cases and 466,003 deaths were reported in 2020, both sexes, all ages [2]. The rates of PaCa incidence and mortality vary across countries, gender, age, and environmental factors. It is worth emphasizing that the published figures for PaCa vary in several recent publications, as there is an uptick in PaCa incidences worldwide. For instance, according to Stoffel et al. [3], PaCa is the seventh leading cause of cancer-related fatalities while according to Olajubutu et al. [4] and their relevant references, PaCa is rapidly turning into a global threat and it is projected to be the second most common cause of cancer mortality by 2030 in the United States [5]. Unquestionably, as PaCa is frequently diagnosed too late to be treated effectively, it is no exaggeration to say that the mortality is similar to the incidence, because of the poor prognosis of this malignancy [6]. As a consequence, it is believed to be a leading cause of cancer-related fatalities with projections in the coming future [7]. Adenocarcinomas are by far the most prevalent form of PaCa and originate in exocrine cells (cells responsible for producing digestive enzymes) [8].

PaCa, or more specifically pancreatic ductal adenocarcinoma (PDAC), may not cause early signs or symptoms, and, as we already mentioned, the delayed diagnosis is the main cause of the inability to cure patients. Therefore, for any attempt to eliminate this disease, one of the primary challenges continues to be developing more efficient early detection methods and approaches to stop patients from being tardily diagnosed and treated. Unfortunately, in current clinical practice, there are still no satisfactory techniques available for early detection [9]. Most of the conventional imaging modalities such as magnetic resonance imaging (MRI), computed tomography (CT), positron emission tomography (PET)-CT, or ultrasound have difficulty in detecting small, early-stage, or premalignant lesions due to the deep anatomical position of the pancreas. Consequently, any novelty attempts for early diagnosis and treatment of cancer (e.g., nanomedical theranostics) are obviously very crucial to illness prognosis. To overcome the lack of early diagnosis problems, Lu et al. [9] developed a high-resolution three-dimensional endoscopic optical coherence tomography (OCT) system that functions in both diagnosis and treatment in a minimally-invasive mode. According to Lu et al. [9], their system (tested in phantom and ex vivo, in specimens resected at surgery from patients) can accurately find and assess the location and size of the early-stage PaCa or precancerous lesions and, then, treat them from within the pancreatic duct, using high dose rate brachytherapy. Additionally, another promising emerging tool that extends beyond conventional diagnostic practices is radiomics, which leverages digital images obtained from routine imaging modalities to extract data and derive quantitative insights into characteristics such as the heterogeneity of tumors, thereby enabling preoperative prediction of the stage of the disease, that can help decide the clinical treatment strategy. Taking advantage of this, Ren et al. [10] developed a radiomics model that could differentiate between early- (I–II) and late-stage (III–IV) PaCa based on characteristics derived from the CT images of 71 patients with this disease, which demonstrated a high discriminatory capability.

**Figure 1:**
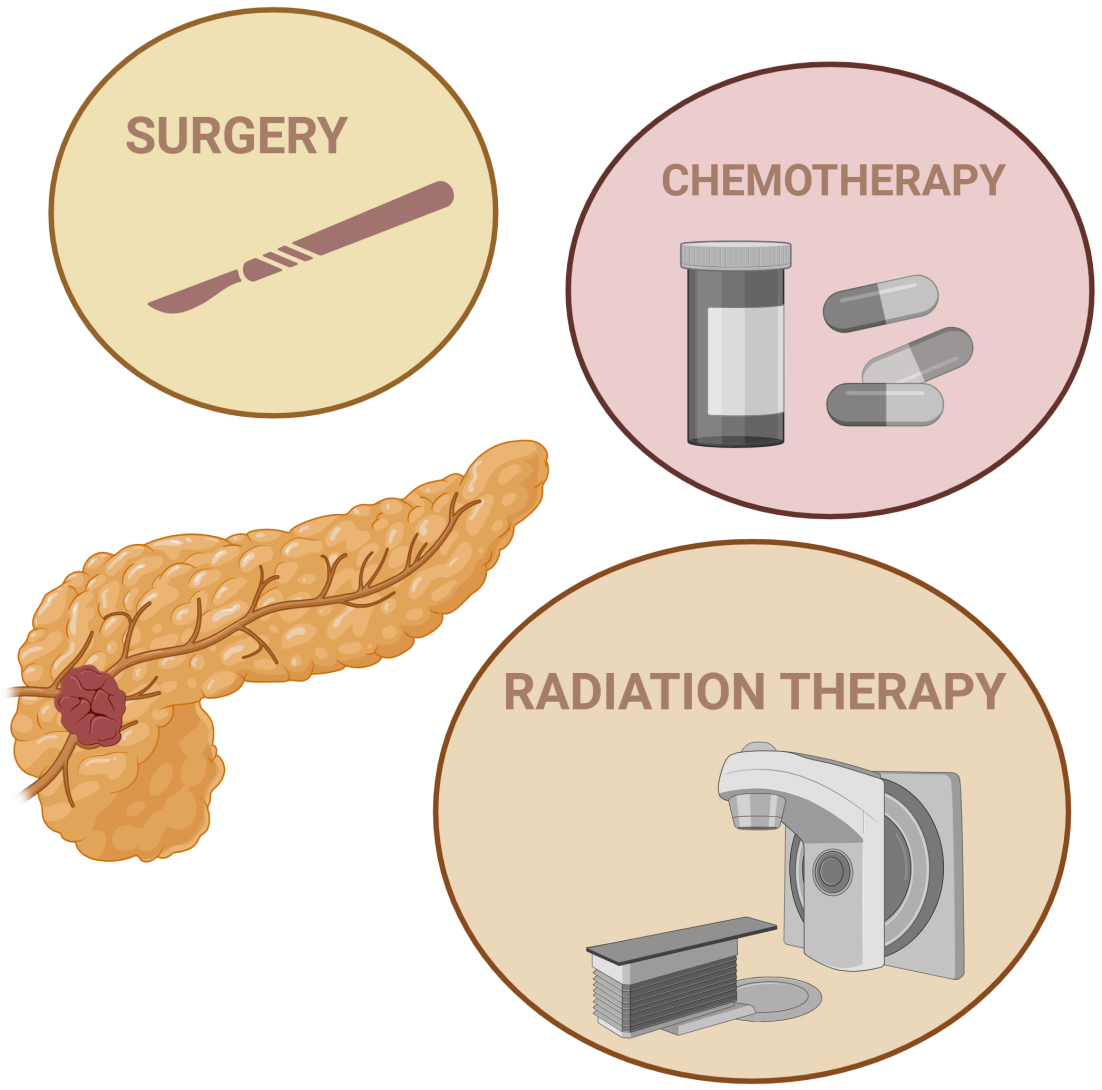
**Conventional treatment approaches for pancreatic cancer.** Created in BioRender. (2025) https://BioRender.com/04rec1z

It is commonly agreed that the three fundamental cornerstones of oncology care are surgery, radiation, and chemotherapy (as shown schematically in Figure 1). As radical surgery is considered the pillar of PaCa therapy, the classification of PDAC, a disease that develops in the pancreas’ exocrine region, usually presents the following levels, according to MD Anderson Cancer Center: resectable (R-PDAC), borderline resectable (BR-PDAC), and unresectable locally advanced (LA-PDAC) [11, 12]. The main reason that PaCa is considered unresectable is the tumor involvement with or in proximity to major blood vessels (e.g., the superior mesenteric vein, the superior mesenteric artery, the portal vein, and the gastroduodenal artery). Pancreatic tumors can grow in the ducts, tail, or head of this organ, and are often unreachable for removal by traditional surgery [13]. Several surgeons consider that for regionally advanced PaCa, which includes the encasement of critical vascular structures, surgical resection is not an option [6]. Nevertheless, resectability depends on the perspective of the surgeon, as very aptly reported by Giannone et al. [14] in their multicenter, blinded, prospective assessment of interobserver agreement on National Comprehensive Cancer Network (NCCN) resectability status classification for PaCa. Otherwise, the operability status should be assessed by a multidisciplinary team following an evaluation with high-quality cross-sectional imaging [15–17]. In a recent report, Mizrahi et al. [15] presented the resectability criteria for BR-PDAC according to MD Anderson Cancer Center, to the NCCN and the International Consensus 2017 based on Eastern Cooperative Oncology Group. Unfortunately, most patients are usually diagnosed at advanced stages with disease that cannot be resected, with metastasized PaCa cells in some cases, which makes the surgical operation impossible. For patients with localized disease, a multidisciplinary strategy is essential to enhance survival and improve outcomes [18–20].

However, survival is not the sole outcome to be considered, but quality of life must also be assessed, both physically and emotionally, then [21] and in our days [20]. No matter that the main purpose of any theranostics intervention is to influence survival, but, as a great number of patients experience a painful condition during their end-life, the quality of life is also an important factor.

The aim of our work is to evaluate the possibility of nanomedicine, which employs a diverse range of nanocarriers, such as liposomes, polymeric nanoparticles (NPs), micelles, gold NPs (GNPs), and quantum dots in PaCa therapy [22–24], by mixing different scientific cultures, a multidisciplinary approach to the treatment of the disease is sought, mainly studying the recent available literature.

## PaCa: a disease with few therapeutic options

In oncology, the desired therapeutic effect against cancer can result from direct tumor cell death via necrosis or apoptosis, vascular damage causing tissue ischemia and subsequent cell death, immune modulation, or a combination of these factors [25]. Although there are several strategies against PaCa—including surgery, radiotherapy (RT), chemotherapy (Figure 1), and in recent times targeted immunotherapy [26], PaCa remains one of the deadliest cancers in the world and one of the most difficult to treat.

According to NCCN guideline [27], the sequence of available treatments is:

- Neoadjuvant (pre-) treatment is administered before surgery for tumor shrinkage. This therapy can help transform a borderline tumor into one that is resectable.
- Primary treatment typically involves surgery for PaCa patients. It is the primary approach for resectable cancer.
- Adjuvant (post-) treatment is given after surgery to eliminate any remaining tumor or cancer cells. This therapy is also effective for cases of recurrence.

Over the past decade, multimodal treatments have played a significant role in improving the prognosis of this disease. Moreover, outcomes continue to improve, and promising new treatment options have emerged in recent years, alongside the proposed strategy for developing new cancer nanomedicines. It seems that, soon or late, the best care for patients with PDAC, being among the most lethal of human cancers, would require a multidisciplinary approach [18, 20, 28, 29].

Definitely, it seems that chemo- and radio-therapies have limited specificity, significant systemic side effects, and low efficacy in treating PaCa. Let’s try to discern the possible reason(s) for this behavior. A factor that makes PDAC more difficult to respond to conventional treatments is that this tumor is marked by the formation of a particularly dense fibrotic stroma, consisting of both cellular and non-cellular elements such as pancreatic stellate cells (PSCs), that differentiate into heterogeneous fibroblastic cells, type I collagen, immune cells, adipocytes, and hyaluronan [30]. This complex micro-environment plays multiple roles in regulating tumor growth and response to therapy [31]. The activation of fibroblasts and the increased synthesis and accumulation of extracellular matrix (ECM) proteins and collagen induce a desmoplastic reaction in pancreatic tumors, which is also referred to as desmoplasia [32]. Desmoplasia, a fundamental characteristic of PDAC, originates from the Greek words desmos meaning “band” or “fastening” and plassein which is to “form” or “mold” [33]. The desmoplastic fibrotic stroma, in combination with the characteristic rapid proliferation of cancer cells and inadequate vascularization, influences the tumor oxygenation levels, inducing hypoxia [34]. In the recent review of Tao et al. [34], the interested reader could find details on the molecular mechanisms underlying hypoxia, in both pancreatic cancerous and stromal cells, focusing on novel therapies targeting the tumor hypoxic microenvironment itself. In the last years, there has been growing interest in the tumor microenvironment (TME) of PaCa and several indications dictate that the PaCa microenvironment also plays a role in metastasis, making it a potential target for combination therapy aimed at improving overall survival [35].

In a relatively recent review, Anderson et al. [5] summarized in a table a list of clinical trials involving agents targeting the PDAC TME. They report that the TME and specifically the tumor stroma, offer promising targets for combination therapies, which include chemotherapy, radiation, immunotherapy, and/or targeted therapies in patients with PDAC.

In the armamentarium of the therapeutic strategies for PaCa management approaches, we distinguish systemic action methods (e.g., chemotherapy, immunotherapy, hormone therapy) and loco-regional procedures [e.g., open or minimally invasive surgery for removal of resectable cancerous tissue, targeted RT, brachytherapy, photothermal therapy, photodynamic therapy (PDT)] and, of course, treatments to control symptoms.

The Whipple procedure, also known as pancreaticoduodenectomy, is the standard surgical technique used to treat tumors located in the head of the pancreas but presents a high rate of difficulties, complications, and risk incidence. As in other pathologies involving surgical operations, the interest in minimally invasive surgical procedures (e.g., laparoscopy and robotic surgery) has increased substantially in recent years in PDAC [20, 36]. Apart from surgical resection of PDAC, RT is a loco-regional cancer treatment procedure that uses high-energy ionizing radiation, either in the form of electromagnetic waves (like X-rays and γ photons) or particles (like alpha-particles, beta-particles, carbon ions, protons), to kill cancer cells (by damaging their DNA or by producing free radicals and stopping cancer cells from growing and spreading). The various radiation techniques, which have been used in the treatment of locally advanced PaCa, are classified as three-dimensional conformal RT, intensity-modulated RT (IMRT), stereotactic body RT (SBRT), and intraoperative RT. By attending the mode of the ionizing radiation application to PaCa tumor masses, suitable interventions include external-beam RT and internal-beam or brachytherapy (radioactive materials applied directly on the tissue being treated).

Regarding chemotherapy, the patient is given specific cytotoxic drugs orally or intravenously, acting on the whole body and on the tumor. Nevertheless, the majority of the chemotherapeutic drug regimens induce several side effects in patients, with marginal and transient improvements at best to their PaCa, as resistance mechanisms often prevail [29]. In the last years, chemotherapy drug combinations were developed, aiming to the increased survival of patients. In this case, two or more chemotherapy drugs are typically given to treat patients, depending on their ability to tolerate therapy. Chemotherapy can be used as a single treatment in patients who are planning to be operated (neoadjuvant) to shrink the tumor, or after the operation (adjuvant), or just to increase survival in patients who are not suitable for operation. Certainly, chemotherapy plays an important role but causes side effects.

Currently approved first-line treatments, for advanced disease in patients with a satisfactory response, are combination chemotherapy with either the modified FOLFIRINOX plan (5-fluorouracil, leucovorin, oxaliplatin, and irinotecan) or combination gemcitabine plus nab-paclitaxel, according to findings from the phase 3 PRODIGE 4 and MPACT trials, respectively [37–39]. Nevertheless, modern PaCa treatment usually involves drug combinations, often with divergent pharmacology, and this can make dosing and tumor distribution in synergistic proportions more challenging [38]. Two decades in the past, a phase II multisite study was undertaken by the Hellenic Cooperative Oncology Group, in an attempt to enhance the effectiveness of gemcitabine in patients with advanced, inoperable, or metastatic PaCa. They combined gemcitabine with carboplatin and their results indicated that this addition to standard gemcitabine treatment for advanced PaCa did not improve the overall survival, as the response rate and survival remained inadequately low [40]. Moreover, Cavanna et al. [41] evaluated the efficacy and safety of a FOLFIRINOX regimen in advanced/metastatic PaCa in an Italian general hospital. They analyzed retrospectively the medical records of 50 consecutive patients with advanced (13 patients) or metastatic (37 patients) PDAC, 18 patients receiving a full dose compared with the 32 patients receiving an attenuated dose of FOLFIRINOX as first-line chemotherapy, between July 2013 and July 2017 [41]. They concluded that the FOLFIRINOX dose reduction, as performed in their patients, did not compromise efficacy, while reducing the number of adverse events.

In present-day oncology, with the possibility of detecting target mutations in the genome of cancer cells, novel therapies have been developed that target these mutations, in the sense that they bind to their protein products and bring about a therapeutic effect. In the case of PaCa, such molecules have only been tested in the last decade and seem to show clinical benefit, especially at a time when this type of cancer is considered as one of the hardest to treat, with relatively few therapeutic options to date. However, the data is constantly changing.

At the present time, it is known that treatment with poly(ADP-ribose) polymerase (PARP) inhibitors (e.g., olaparib) shows benefits in patients who have a mutation in DNA repair genes (*BRCA1*/*2*, *PALB2*) and have previously received platinum-based chemotherapy [42]. At the same time, when cancer cells show microsatellite instability-high (MSI-H) or high tumor mutation burden (TMB) they may have long-term benefits from the administration of immunotherapy with PDL1 inhibitors (pembrolizumab) [43]. Recent data from clinical studies (basket trials), regarding rearranged during transfection (*RET*) fusion tumors, show that the administration of the agent selpercatinib confers clinical benefit [44]. Similarly, neurotrophic tropomyosin receptor kinase (*NTRK*) fusion tumors, including those of the pancreas, have shown sensitivity to the inhibitors of the corresponding kinase larotrectinib/entrectinib. The *RAS-G12C* mutation in PaCa cells has predictive value for the response to treatment with the agent sotorasib according to phase I/II clinical studies (the drug is already an indicated treatment for lung cancer). Finally, from December 2024, patients with neuregulin 1 gene (*NRG1*) fusion PaCa can receive the agent zenocutuzumab-zbco in disease that is resistant to previous lines of treatment [45]. It should be noted however that the probability of finding such mutations in PaCa cells is very small, and it is now imperative that each patient undergo a complete screening for mutations in the tumor genome in order to identify all available therapeutic options where they exist.

Recently, Kolbeinsson et al. [18] published a short overview of current practices in PaCa treatment, along with emerging therapies and novel multimodal treatments that are underway. Innovative targeted therapies, based on genomic results could potentially improve survival and quality of life in PDAC patients [46]. Although multimodal treatments have helped improve the prognosis of this disease, even though long-term outcomes remain unsatisfactory [47]. In 2023, in an editorial report of “*World Journal of Gastrointestinal Surgery*” Damiano Caputo offered an overview of PaCa management, covering the current status of multimodal therapies and the growing immediate need for the development of early diagnostic tools for PaCa [7]. Caputo affirms as a concluding assumption to this special issue that quality studies in the fields of basic, clinical, preventive, and transnational medicine will provide additional support for research in the fight against PDAC. A comprehensive literature review was published in 2024 by Wang et al. [48], in which they present an outline of current treatment modalities for PaCa, focusing on clinical advancements after a thorough search in databases (e.g., PubMed, MEDLINE, and clinical trial registries).

## Modern approaches of nanomedicine in PaCa theranostics

Emphatically, PaCa, often accompanied by the expression a “silent killer” disease, is not a rare disease anymore and requires urgent attention. Beyond any doubt, as we already referred, the increasing cancer burden drives global research to shift from monotherapies with systemic effects to multimodal targeted and personalized treatment strategies [49]. In a recent review article, Jaidev et al. [50] discussed multiple therapeutic and theranostics nanocarriers for PaCa. They highlight the advantages of developing specialized drug delivery carriers for treating high-mortality cancers like PaCa. Theranostics NPs represent a new class of delivery carriers, designed to carry both diagnostic and therapeutic agents [50]. A decade ago, Esposito et al. [51] summarized in a review the knowledge that covers both well-established concepts and recent advancements in cancer diagnosis and treatment, emphasizing the cooperation between nanotechnology and targeting to pave the way to personalized tumor therapies. The aim of their overview was the exploitation of novel, at that time, promising processes and the feasibility of introducing nanotechnology/nanomedicine into PaCa theranostics procedures, as this field has grown tremendously in several biomedical disciplines in the past two decades and, consequently, we hope to “buy time” for PaCa patients, succeeding early diagnosis and treatment with each coming year.

First, in what follows, we try to explain the concept of “nanotechnology” and its progress over the past decades, particularly in the concept of cancer nanomedicine. Nanotechnology was initially introduced in 1959 by Richard Feynman during his renowned Caltech lecture, “There’s plenty of room at the bottom.” [52]. Since then, advancements in nanotechnology have been evident across various fields, including clinical applications [53]. The next step is to discuss what requirements nanotechnology must fulfill for the application in oncological systems to speed up and make care safer, and what nanosystems (e.g., NPs) we could use, to be easy and safe to handle.

According to Stylianopoulos et al. [32], “the spread of a drug throughout the body and its delivery to the tumor (pharmacokinetics) depend on its physical properties, including size, shape, charge, binding affinity, and metabolism or degradation”. In their feature review, Stylianopoulos et al. [32] show that physical forces have a crucial role in tumor progression and cancer treatment and, therefore, the application of principles of engineering and physical sciences to oncology (physical oncology) forms a multidisciplinary field that provides powerful insights into the mechanisms by which these forces influence tumor progression and contribute to resistance against the delivery and effectiveness of molecular, nano-, cellular, and immuno-medicines. Furthermore, to achieve more favorable clinical outcomes of nanomedicine, novel and notable studies that offer valuable insights and a deep understanding of the physical phenomena involved in the in vivo behavior of nanomedicine may improve next-generation NP designs [54].

## NPs and nanomedicine, with emphasis on PaCa

Nanotechnology, derived from the Greek word for “dwarf” (nános), refers to physical dimensions of 10^–9^ m or 1 nanometer (nm) typically extending from 1 nm to 100 nm. NPs currently in clinical trials, or approved for such trials, vary in size from 1 nm to 200 nm. In fact, nanotechnology concerns systems of different schemes and geometries (e.g., nanospheres, nanotubes, nanoshells) that are nanometer in size in at least one dimension, representing a dynamic research field and a techno-economic domain with wide expansion across numerous application sectors [55]. NPs of different schemes exhibit a high surface to volume ratio, making them highly reactive. The term “nanoparticle”, in its common usage, does not include nano-dimensional substances found naturally in nature (e.g., viruses, DNA double helix).

The application of nanotechnology in medical sciences, referred to as nanomedicine [56], particularly in cancer treatment, is a rapidly growing field. Certainly, the advancement of new nanomedicine procedures is attributed both for cancer management and for diagnosis and prevention, i.e. for theranostics applications. For example, considering the toxicity of some already tried chemotherapy regimens, there is a need for continuous research work to find an efficient substitute for chemotherapy and a more effective therapy for cancer. While attempts for cancer treatment and improvement of the drug effectiveness with nanotechnology are still in the research or development phase, NPs have been widely utilized in biomedical applications [57]. However, as Mundekkad and Cho [57] recently affirmed, 25 years after the introduction to the market of the first nanochemodrug Doxil, only a small number of formulations have progressed beyond the clinical phase and much of the plethora of fundamental research results have not yet been translated into clinical applications. Furthermore, the interested reader can search the nanodrugs that have recently undergone clinical trials for PaCa, presented in a table with several data compiled by Mundekkad and Cho [57] from ClinicalTrials.gov. Recently, Nirmala et al. [53] presented a comprehensive review of the origin, history, and timeline of cancer nanomedicine or nano-oncology as one of the various sub-divisions of nanomedicine. Among other aspects of cancer nanomedicine applications, they also reviewed in a table some of the approved drugs in the market and drugs under clinical studies for PaCa treatment. According to Nirmala et al. [53], the amount of nano-formulations under clinical studies has been growing over the past few years with many currently in phase II and III. It appears that nano-oncology is an emerging approach in cancer treatment that includes the utilization of nano-dimensional therapeutic materials as anticancer agents, and it has generated promising results in research and clinical trials [58, 59].

NPs (1–100 nm) can be used to treat cancer because of their unique characteristics like biocompatibility, reduced toxicity, increased permeability, improved stability, precision targeting, and retention effect. Along the time of nanotechnology evolution, many types of nano-formulations including liposomes, metal and metal oxide NPs, polymer-based NPs as dendrimers and micelles, nanocrystal NPs, and lipid NPs have been created and approved for medical use by multiple agencies globally, with many more undergoing various phases of clinical and pre-clinical studies [60–62]. NPs can be administered to an organism by inhalation, by infusion into the blood, or by local deposition (surgically or non-surgically), depending on the location of the tumor. Regardless of their small dimensions, NPs can enclose small pharmaceutical compounds, while their high surface to volume ratio enables the attachment of ligands, DNA and RNA strands, peptides, or antibodies. Their surface modification enhances their functionality, improving therapeutic outcomes and enabling targeted delivery to specific sites [63].

Definitely, nanotechnology in medicine, i.e. nanomedicine, is a rapidly developing area that concerns the application of nanotechnology for:

a) the study of biological systems, of the same order of magnitude as that of basic biological units (cell, DNA, proteins),
b) single-molecule or cell-level interventions (with obvious and unique potential for targeted therapy and diagnosis),
c) development of biosensors and bioactivators for various theranostics applications.

Recently, Kashyap et al. [61] summarized in a comprehensive review various methods in cancer theranostics, highlighting the utilization of the so called smart nanomaterials (such as organic NPs—NPs, inorganic NPs, and carbon-based NPs) on the nanotechnology-based theranostics systems to aid various clinical and preclinical cancer therapies. Moreover, the interested reader can study a comprehensive overview of the role of nanotechnology advancements in the detection and treatment of solid tumors and an outline of the commercially available nanosystems and those currently undergoing preclinical research, in cancer therapeutics [64].

With each passing year, as nanotechnology is already starting to have an impact on the healthcare system worldwide, several papers appeared in the field, either postulating or investigating the promising applications in PDAC combat. For an overview of several implementations of nanotechnology in healthcare and multi-dimensional applications in the modern medical sector, interested readers could refer to the review of Prasad et al. [65]. Furthermore, Li et al. [66] provided some years ago a review of the global cancer nano-theranostics, focusing specifically on their applications for managing PDAC. In their overview, they summarize and present in a table format some exhibits of nanomedicines for the treatment of PaCa, as well as the clinical phase of the related clinical trials. They conclude that, at the time of their publication, the role of loco-regional interventional procedures in advanced PDAC was restricted, although interventional techniques have been successfully used in clinical practice for the effective cure of various cancers [66]. Greene et al. [29] presented in a review several attempts to exploit nanotechnology and NP-based platforms for PaCa therapy, focusing on those studies where the unique features of NPs have been leveraged to override drug resistance.

According to Siamof et al. [67], “the versatility of nanotechnology in cancer requires concerted efforts and interdisciplinary cooperation between scientists, academics, clinicians, and regulatory authorities”. Regarding the regulatory authority involvement, we recall here the EU-funded NoCanTher project that was launched in 2016, with the aim of bringing new nanotechnology therapeutics to the fight against PaCa [68]. The ongoing clinical study was taking place at two sites in Spain, testing the new therapy on patients with locally progressed PaCa. Their strategy, incorporating magnetic iron oxide NPs, allowed heat to be exclusively applied to the area where the NPs were located—in this case, pancreatic tumors—without harming the surrounding healthy tissues [69]. By means of systemic administration, chemo-agents are being delivered throughout the body through the bloodstream, targeting both tumors and healthy organs. In fact, systemically injected drugs are developed to kill also metastatic cancer cells, i.e. cells that have transferred from the pancreas to other organs. When local interventions are used, the chemotherapeutic agents (including injectable, implantable, and micro/nano-based formulations) are kept specifically at the tumor site, minimizing unwanted toxicity [70, 71].

Recently, Lambin et al. [72] summarized the locoregional therapies and their primary impacts on the TME of PDAC, emphasizing the TME characterization for ongoing clinical trials. They present in a table the ongoing studies that explore locoregional therapies either as standalone treatments or combined with chemotherapy or immunotherapy for PDAC [72]. Locoregional treatments, including hyperthermia (HT) with microwave ablation or radiofrequency ablation (RFA), RT, irreversible electroporation (IRE), and high-intensity focused ultrasound (HIFU) therapy, are receiving growing interest for their capacity to precisely aim the tumor while minimizing harmful systemic side effects. These therapies can frequently be used in conjunction with anticancerous drugs [70, 73]. Moreover, Wang et al. [74] exploited a biomimetic NP-based platform to enhance the chemotherapeutic scheme in PaCa combat, the mFOLFIRINOX regimen, synthesizing the so called CNP@folfirinox NPs. The primary components of the mFOLFIRINOX regimen, including 5-fluorouracil, oxaliplatin, and irinotecan, were encapsulated in poly(lactic-co-glycolic acid) (PLGA) to form the core of NPs. These NPs were then coated with the PaCa cell membrane (PCCM), namely CNP@folfirinox, to enhance targeted delivery to PaCa while avoiding immune detection and attack [74].

Magnetic NPs represent another widely utilized class in the field of theranostics due to their favorable biocompatibility and low immunogenicity. Their surfaces readily accommodate the attachment of ligands for targeted therapy, while their intrinsic magnetic properties provide strong contrast in MRI. Consequently, these NPs have garnered significant interest among researchers. For instance, Ren et al. [75] successfully developed theranostic PEG-coated, Cy-7 functionalized, EMO-loaded Fe_3_O_4_ multifunctional nanoplatforms and validated their efficacy through in vivo experiments in mice, where they showed good results for the PaCa cell treatment and MRI imaging, that could also be used both in the detection and treatment of the disease.

Several nano-formulations have progressed to clinical evaluation in PaCa patients and some of them have been approved for clinical application so far, either by the FDA in the United States or the European Medicines Agency (EMA) in the European Union. For example, Abraxane, an albumin-bound NP formulation of paclitaxel, received FDA’s authorization in 2013 as a first-line therapy of metastatic PaCa combined with gemcitabine [29]. Furthermore, irinotecan-loaded PEGylated liposome (Onivyde) and cytarabine-loaded liposome (DepoCyt) were also approved for the treatment of PDAC and lymphomatous meningitis, respectively [76].

**Figure 2:**
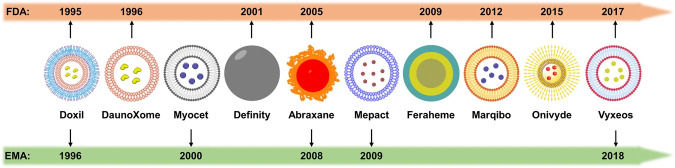
**Timeline of the advancement of smart nanoparticles for cancer diagnosis and treatment.** Reprinted from [77], CC BY

Apart from conventional NPs, some evidence appeared in the relevant literature on the so called smart NPs, which can respond to biological signals or be guided by them, acting as a promising drug delivery platform for precise cancer treatment [77]. In their publication, Sun et al. [77] present several clinically approved smart NP formulations, approved either by the FDA in the United States or the EMA in the European Union, in a timeline reproduced in Figure 2. Undoubtedly, by ensuring that NPs are transferred only to the tumor mass, we can increase the treatment selectivity.

Modern cancer diagnosis and treatment usually involves amalgamations of drugs, often with diverse pharmacology, and this can make dosing more complex and tumor distribution in synergistic proportions [78].

## Using radiation-based therapies for PaCa, in connection with nanomaterials

Radiation-based therapy, is a broad term, referring to the medical use of both ionizing electromagnetic radiation and/or non-ionizing radiation in cancer treatment to manage malignant tumors. One of the problems in the field of RT (internal and external beam RT) is the treatment of deep-seated tumors, like PaCa, without harming the surrounding healthy tissues. The mass-less and charge-less γ and X-ray photons are very penetrating and transfer their energy to the entire volume of tissue irradiated. However, most of the radiation is only delivered to a depth of 0.5 cm to 3 cm beneath the patient’s skin, relying on the initial incident energy. So, the beam gradually loses its energy until it reaches the malignant pancreatic tumor target. The development of high cutting-edge technology in recent two decades has allowed the amelioration of new methods for the treatment of cancer, both at the systemic level and in targeted interventions, such as immunotherapy, targeted forms of ionizing RT (e.g., SBRT, proton therapy, brachytherapy) and non-ionizing radiation-based therapy, such as PDT and HT. According to Cao et al. [79] published results in 2023, RT holds promise as a treatment for initially inoperable non-metastatic PaCa, resulting in improved local control. Nonetheless, additional research is required to establish the optimal dose and evaluate the effect of RT on survival outcomes. Recently, Tremi et al. [80] reported an overview showing the ongoing need for more systematic studies prior to the application of metallic-based NPs in medical practice, for improvement of the effect of ionizing RT. They tabled some clinical trials already implemented with combined NP-radiation therapies in cancer patients. Their table includes currently undergoing or completed clinical trials (not yet recruiting, recruiting, active, completed) in several target tissues and applications, including pancreatic and lung cancer.

**Figure 3:**
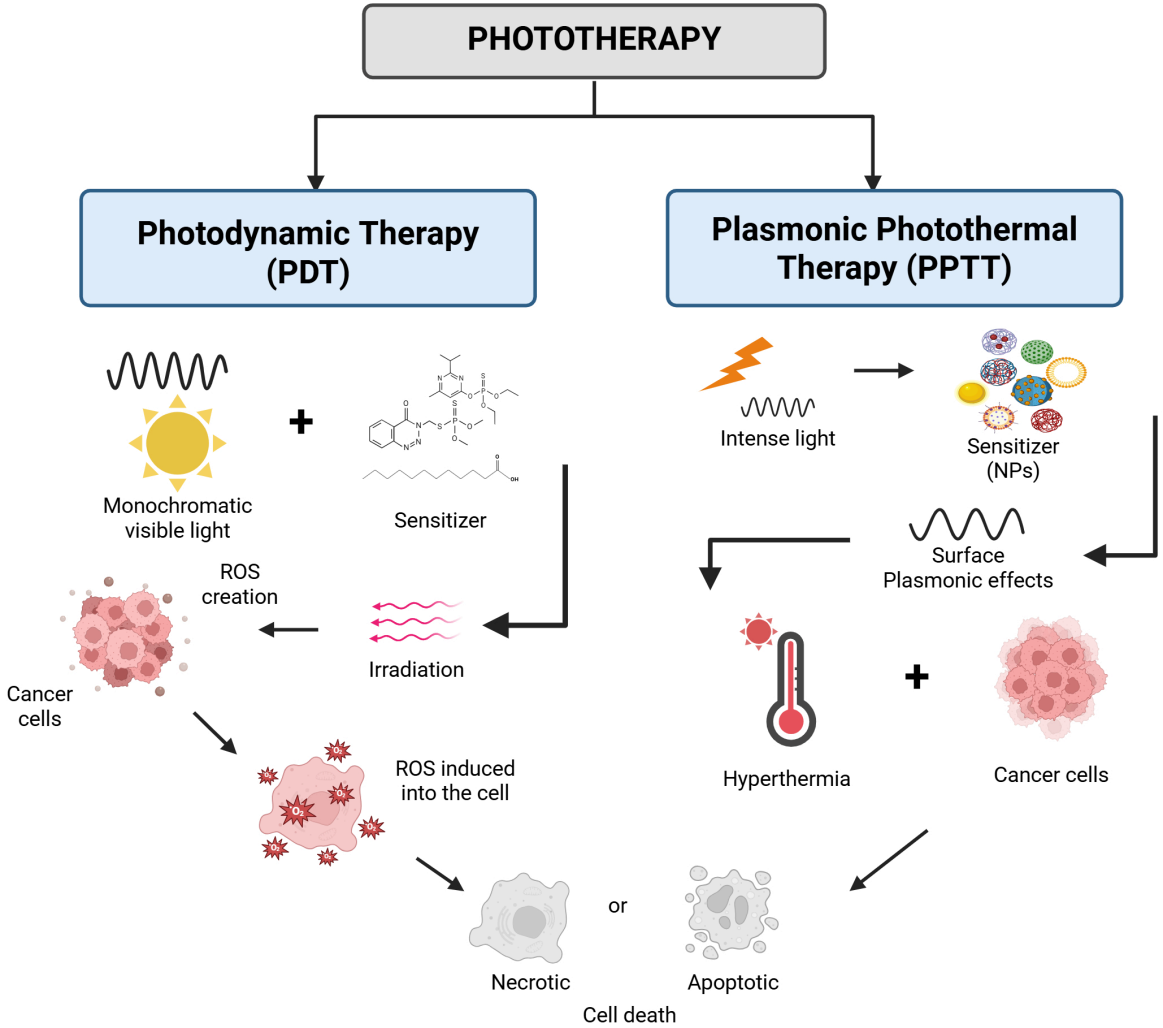
**Schematic representation of the two main types of non-ionizing, monochromatic light therapy (phototherapy), namely the photodynamic and the plasmonic photothermal therapy of cancer.** NPs: nanoparticles. Created in BioRender. (2025) https://BioRender.com/9keqyrb

Apart from ionizing radiation applications in cancer diagnosis and therapy, the development of non-ionizing electromagnetic radiation changed dramatically the landscape of the anti-cancer fight. The laser light (coherent radiation in the optical part of the electromagnetic spectrum) can be used either to surgically remove cancerous and precancerous growths or for symptom relief. It is generally recognized that using laser instead of standard surgical tools in surgery can minimize bleeding and normal tissue damage while preserving a lower infection risk. Among the different laser-tissue interaction mechanisms, photothermal ablation was only considered in the management of PaCa, reported in a up to 2018 literature review of Saccomandi et al. [81]. They present in their review a number of ex vivo, preclinical, and clinical applications of several thermal ablation-based therapies (12 papers included in the review being based on laser ablation), as well as NP-mediated laser ablation of PaCa. Unfortunately, despite the revolution brought in oncology by the various applications of lasers and because the location of PaCa in the body requires proximity of the laser beam transport medium to the target (e.g., with optical fibers), the dosimetry and the overall application control become difficult for effective PaCa resection [82]. Definitely, laser light can also be employed non-surgically, for PDT and plasmonic photothermal therapy (PPTT) in the treatment of cancer and other diseases [83, 84], as we discussed in the following sub-paragraphs and illustrated schematically in Figure 3.

## PDT as a promising option for PaCa

To describe briefly PDT, we recall that the biophysical principle of PDT is grounded in the concurrent operation of three factors: photosensitizer, oxygen, and light. It is worth mentioning that while each of these three factors is harmless (when alone), their combination results in cell death via a photodynamic process.

Furthermore, we can say that PDT can be regarded as a light-triggered, targeted form of chemotherapy [83]. The light is usually monochromatic non-ionizing radiation [by a laser or a light-emitting diode (LED) source], selected to match the maximum absorption wavelength of the photo-sensitizing non-cytotoxic drug molecules, within the visible or near-infrared (NIR) range of the spectrum [85, 86]. A recently appeared comprehensive review by van Straten et al. [87] results that PDT should have a leading role in cancer treatment, either as an element of a multimodal strategy or as an independent treatment for early-stage disease, supportive care, or rescue treatment. Certainly, several obstacles still hamper clinical applications, although with the development of new photosensitizers and nanomedicine-based formulations, the current restrictions can be surpassed establishing PDT as a powerful equivalent for conventional treatments.

In the last 20 years, PDT was introduced in pre-clinical and clinical states as a locoregional treatment alternative for PaCa. The first clinical evidence of PDT in PaCa was realized by Bown et al. [88]. They launched the earliest clinical trial of the use of PDT to treat PaCa in 2002. Their original article reported a phase I study of PDT for PaCa treatment. Moreover, they mentioned that, while the majority of research on PDT has been focused on lesions in the hollow organs’ walls or on the skin, there has been a growing interest in further exploring its potential for the treatment of solid organs’ lesions such as the pancreas [88]. Their study involved 16 patients with unresectable pancreatic head cancer, that underwent mTHPC-mediated percutaneous interstitial PDT (I-PDT), resulting in significant tumor necrosis on a post treatment image examination. Their work was also cited by Karamanolis and Mallas [89], in their editorial paper in 2004. In 2014, people from the group of Bown published a study to evaluate the safety and the effectiveness of a next generation photosensitizer, employing a formal dose escalation method to identify to what extent the necrosis changed in relation to the delivered light dose. They found no significant adverse effects utilizing PDT either pre or post other oncological treatments, although their protocol required at least a timeframe of a month between treatments [90].

Conventional PDT is typically a non-invasive approach to cancer treatment. Nevertheless, a concern for human application in PDAC is the fact that PaCa is a bulky, deeply located solid organ tumor, resulting in limited light penetration into the cancerous volume. Consequently, apart from external beam PDT (applied mostly in dermatology and other superficially located tumor masses), intra-tumor light delivery is also possible in a semi-invasive procedure (I-PDT) [91]. In I-PDT one or more laser fibers are inserted into the target tissue, to activate photosensitizers in deeply seated tumors or tumors that are more than 10 mm in thickness. The laser fibers can be inserted via needles, or placed in catheters [92].

An additional obstacle for PDT clinical applications is the fact that conventional photosensitizers are hydrophobic agents that generally demonstrate low bio-availability. Moreover, photosensitizers tend to cluster easily in aqueous solutions, a phenomenon that can impact their photophysical properties (reducing singlet oxygen formation), chemical properties (lowering solubility), and biological properties (biodistribution and pharmacokinetics) [93]. To overcome this, using NPs as transport vehicles for conventional photodynamic agents was proposed. Hafiz et al. [94] reported the experimental results of a liquid metal-based PDT drug delivery platform, designed for the treatment of PaCa.

The need for well-designed experimental studies, as well as for more clinical studies that further improve PDT, is recognized in several scientific reports (e.g., in the Review article [95]). In this review, the mechanisms of pancreatic tumor cell death or destruction induced by PDT are also schematically presented. Furthermore, several reviews appeared in the specific literature implying that PDT plays an emergent role in the clinical control of PDAC, involving the possibility of combining it with other targeted agents [31]. Considering the recognized role of stroma in PDAC progression and its function as an obstacle to drug delivery, Karimnia et al. [31] discussed in the above-cited report that multiple lines of evidence indicate that PDT could have a potential role in this context. Very recently, Yang et al. [96] presented an overview of the existing experimental data on the design, methodology, and oncological outcome of the novel NP-based PDT of PaCa. In this overview, they highlighted the benefits of NPs in enhancing the therapeutic effectiveness of PDT for PaCa treatment and discussed that the current clinical studies along with ongoing phase II/III studies of PDT in PaCa provide a strong foundation for developing NP-based PDT against PaCa. They also highlight the challenges and prospects for the development of NP-based PDT in humans, although transitioning this therapy from laboratory research to clinical application is not without difficulty [96].

Delivering light to internal structures, such as the pancreas, necessitates precise treatment planning and dosimetry, however, pioneering solutions have been developed and clinically proven. Wolfsen [97], in an editorial report entitled “Photodynamic therapy for pancreatic cancer: let’s get serious”, recognizes that the untapped potential of PDT lies in the intricacies of the photodynamic reaction and the challenge of finding the ideal dose that effectively destroys targeted dysplastic and neoplastic tissues while minimizing harm to the surrounding normal tissue. In general, there is a well-founded concern about dosimetry in PDT, which ultimately leads to a concern that clinical adoption of this therapy will be hindered as monitoring and control of drug-light dosage becomes more complex. Unfortunately, a major issue that has arisen in PDT clinical trials is the lack of widespread adoption of this promising practice by practitioners [98].

## Photothermal therapy of PaCa and the role of NPs in the optimization of the treatment

**Figure 4:**
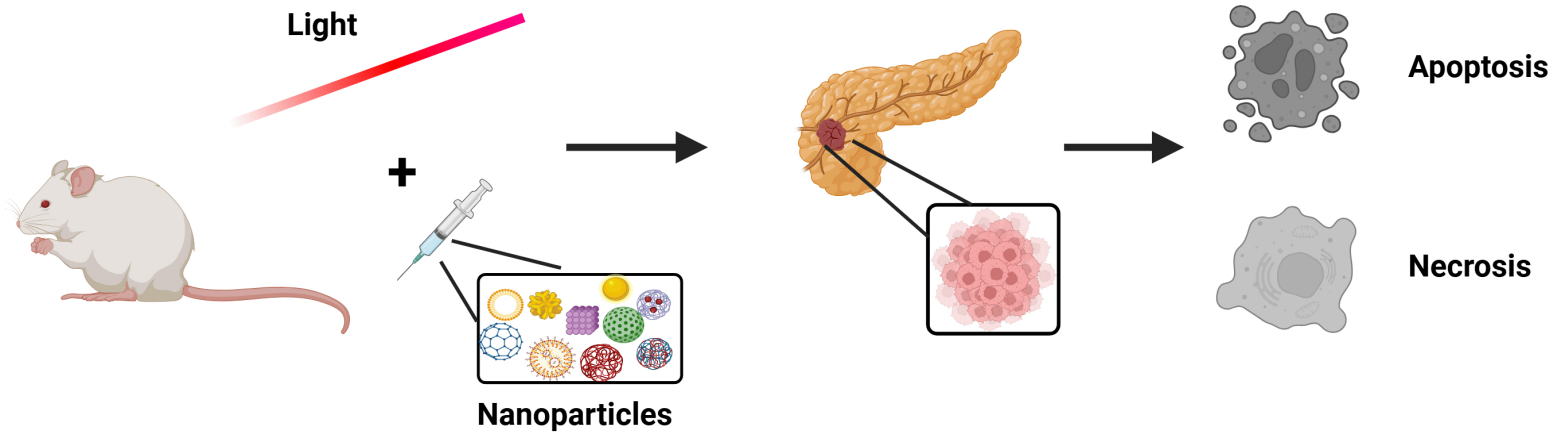
**Schematic representation of photothermal action enhancement, with the use of nanoparticles that will trigger apoptotic or necrotic processes ultimately resulting in the destruction of pancreatic tumors.** Created in BioRender. (2025) https://BioRender.com/9h4bpxn

Photothermal therapy is one of the oldest therapeutic modalities, dating back centuries. Even the father of medicine, Hippocrates, had acknowledged its value, stating in Aphorism (Hp. Aph. 7.87) that “Those diseases which medicines do not cure, iron cures; those which iron cannot cure, fire cures; and those which fire cannot cure, are to be reckoned wholly incurable” [83]. It is based on HT, namely the temperature increase that will lead to apoptotic or necrotic procedures and, hence, tumor destruction (Figure 4). Thermal treatments are a type of medical modality used for cancer therapy, which aims to selectively destroy tumors by increasing locally their temperature until necrosis and/or apoptosis arise. Thermal treatments have gained vast attention as non-invasive or minimally invasive methods for treating focal malignant diseases by using thermal energy sources, including radiofrequency waves, microwaves, NIR laser radiation, and high-intensity focused sonography [13, 99, 100]. Especially in cancer photothermal therapy, the employment of GNPs favors plasmonic effects and enhancement of photothermal action, modifying accordingly their optical and thermal properties. GNPs display distinct and adjustable optical properties as the result of the surface plasmon resonance (SPR) phenomenon [101]. Surprisingly, the properties of gold metal undergo considerable changes as the size is decreased to the nano-scale (1–100 nm) [102]. According to Asadi et al. [100], the explanation of the physical mechanisms behind heat generation in tissues embedded with GNPs, for the overall thermal outcome prediction, is based on both the GNP’s optical properties and the laser irradiation settings, specified to treat the tumor.

Recently, we searched in silico the possibility of utilizing PPTT, in combination with GNPs, to maximize the photothermal effect in inoperable PaCa by simulations using the COMSOL multiphysics software. Therefore, based on Mie theory, we first calculated the absorption spectra of gold nanospheres of different dimensions, in relation to the wavelength of the incident laser radiation. Then, different combinations of laser-NP irradiation parameters were examined through simulation, for the optimal treatment conditions to be established, i.e. for the enhancement of the photothermal effect. Results showed that photo-thermally induced HT in the center of the target volume can be achieved (temperatures up to 45°C), using gold nanospheres with 20 nm diameter, uniformly distributed in the target volume, by selecting the laser emission wavelength that aligns with the peak absorption of the NPs and appropriately adjusting their concentration with the laser power. More studies like this should be encouraged, both in silico and in situ, for the utilization of GNPs as enhancement factors of HT phenomenon, especially on intractable diseases such as PaCa [103].

In addition, the development of biophotonics and nanotechnology offers significant hope for optimizing targeted therapeutic interventions, regarding photothermal diagnosis and synergistic treatment. In fact, many studies lately have been focusing on modifying NPs’ properties to target specific types of cancer tumors, such as PaCa cells, to presume upon the properties of GNPs and the heat generation effect together with possible synergetic treatment actions. For instance, Guo et al. [104] used gold nanorods that were modified using erythrocyte membranes, to become a promising target nanocarrier for gene therapy, exploiting also their properties to enhance heat generation locally in the tissue, when combined with NIR laser light activation. On the other hand, Meng et al. [105] developed Dox@TPAu GNPs capable of selectively accumulating in the mitochondria of cancer cells, effectively overcoming the physical barriers posed by the pancreatic TME. These NPs also exhibit high photothermal conversion efficiency, highlighting their potential as a promising nanocarrier for combined chemo-photothermal therapy [105]. On the same wavelength, Li et al. [106] developed a liposomal gold nanocage (MGL) characterized by a flexible liposomal shell that facilitates efficient intracellular delivery to cancer cells. Upon photoactivation, the nanocage releases maleimide, which depletes intracellular glutathione (GSH), thereby increasing the cells’ susceptibility to oxidative stress. This process induces immunogenic cell death, disrupts cancer cells’ immune evasion mechanisms, and promotes T cell activation. As a result, the treatment not only leads to the destruction of cancer cells but also contributes to the reprogramming of the TME [106]. Chen et al. [107] developed a B7H3-targeted gold nanocage platform (B7H3/Dox@GNCs) capable of releasing doxorubicin in a pH-responsive manner within the acidic TME, thereby enhancing drug delivery to B7H3-overexpressing cancer cells. This nanocarrier effectively induces cell cycle arrest and apoptosis, while concurrently disrupting tumor-associated vasculature and stromal components. By integrating chemotherapeutic and photothermal modalities, the dual-functional system offers a highly specific and low-toxicity therapeutic strategy. Although initially designed for the treatment of non-small cell lung cancer, this approach holds promise for broader applicability in other malignancies expressing B7H3 [107].

## Conclusions

The prospects for controversies over nanotechnology are highly dependent on the uses to which it is put [108]. In the last decade, the rapid advancement and maturation in nanotechnology field is a continuously increasing domain and, therefore, in the coming modern anti-cancer era we can expect that PDT will become ever more convincing. At the beginning of this decade, Huang et al. [93] published a review addressing the question “Can nanotechnology potentiate photodynamic therapy?”. According to the authors, the answer in some areas was an unambiguous positive, but in other areas, it remains uncertain [93].

PDT and nanomedicines that facilitate PDT in different pathologies were recently reviewed by Shah et al. [109], providing a comprehensive overview of the state of the art of in deep-tissue excitation and examining the potential of translating such excitation mechanisms into clinical applications. Significant progress in state-of-the-art activation for deep-tissue PDT includes two-photon PDT [110], upconversion PDT [111, 112], X-ray induced PDT [113, 114], Cerenkov-radiation-induced PDT [114–116].

A very important, but usually underappreciated, aspect of any treatment and obviously of the PDT is the objective dosimetry of any treatment constituent (drug and light dosimetry) [98, 117]. Moreover, a combination of phototherapy, i.e. PDT and photothermal therapy with NP-based RT, not only reduces phototoxicity but also offers excellent therapeutic benefits in cancer battle. Moreover, using NPs with RT has shown promise in cancer treatment and shown excellent therapeutic outcomes in clinical trials [118]. During the last decade, Spyratou et al. [119] reviewed several modern advances in the use of GNPs in PPTT, RT and their combination, based on different types of GNPs, irradiation conditions and protocols. They referred to the interaction mechanisms of GNPs with cancer cells via the effects of NIR and IR electromagnetic radiation, also offering information as a starting point for the optimized protocols in PPTT, RT, and PPTT-RT combined treatment configuration.

Definitely, treatment of cancer is one of the most collaborative fields of basic, pre-clinical, and clinical research. Recent years have seen the advancement of new techniques that complement existing methods or even serve as alternative diagnostic strategies derived from the integration of advanced radiation biophysics, biophotonic, and nanotechnology approaches for cancer regulation [84].

More specifically, it is well known that the management and treatment of patients with PaCa is a complex and demanding task, which requires a multilateral and multidisciplinary effort, both in medical specialties and in the areas of biomedical physics and biomedical engineering. The convergence of different sciences aims at a unified approach to PaCa treatment that allows the achievement of the goals of each specialty for early diagnosis and effective treatment, why not in the framework of personalized medicine? Personalized therapy is marking a new era in cancer treatment, although its actual efficiency remains a matter of significant debate [51]. However, personalized and targeted oncology is certainly moving forward and the use of nanotherapeutic platforms-theranostics techniques that are customized to a particular molecular profile of cancer, using highly specialized and sensitive diagnostic devices, is particularly advantageous in most types of cancers [51, 120]. There are several obstacles to the clinical use of NPs as drug carriers, among them we recall that the microenvironment of pancreatic tumor typically demonstrates abnormal vascularity and excessive desmoplasia, which prevents the ability of blood vessels to perfuse the tumor with either chemotherapy drugs or sensitizing NPs designed to shrink the tumor.

According to Olajubutu et al. [4], while NPs show potential as NP delivery systems, only a limited number of them have been employed for the treatment of PaCa in clinical practice. They established and presented in a table of several novel NP systems currently undergoing clinical trials for PaCa therapy [4]. Nevertheless, having an optimistic view, we consider that in the near future, nanomedicine will be widely used in clinical medical applications. As it was reported recently, nanodrug carriers applied to targeted therapy of malignant tumors will be set as a new approach to diagnosis and treatment, and nano gene carriers will play a key role in the advancement of the clinical application of gene therapy [59]. Moreover, computational tools in light—NP interaction modeling could provide the opportunity to maneuver several biophysical parameters of targeted cancer theranostics in cancer battle [112, 121, 122]. Certainly, the use of an appropriate mathematical model, to simulate a new theranostics application, allows the elucidation of all the parameters and the quantifiable factors prior to deciding on a particular pre-clinical or clinical intervention in oncology. In the past few decades, several promising strategies have been proposed and explored in the battle against cancer. Many unconventional approaches lead to the integration of drugs that are based on NPs in patient treatment and care. However, there are still obstacles and limitations, involving regulatory and approval matters, that may delay the progress. Nevertheless, it is hoped that the scientific community, with the joint participation from academics, regulatory agencies, and industrial partners, will be able to bring out new, patient-centric nanodrugs that will accelerate their journey from bench to bedside, thus providing a paradigm shift from conventional tumor therapeutics.

As the long-standing dream in the field of cancer treatment is the ability to precisely treat deep-located tumors non-invasively, it is imperative to find new diagnostic and treatment platforms that lead to this idea. Future efforts should concentrate on both early detection methods advancement of pancreatic carcinoma and localized tumor destruction for lowering the mortality rate.

At the end of the day, upcoming clinical trials are the final step in resolving the feasibility of nanomedicine in PaCa theranostics by determining their true effectiveness and complications, for future approval of clinical use in humans.

## Abbreviations

CT: computed tomography
GNPs: gold nanoparticles
HT: hyperthermia
I-PDT: interstitial photodynamic therapy
MRI: magnetic resonance imaging
NCCN: National Comprehensive Cancer Network
NIR: near-infrared
NPs: nanoparticles
PaCa: pancreatic cancer
PDAC: pancreatic ductal adenocarcinoma
PDT: photodynamic therapy
PPTT: plasmonic photothermal therapy
RT: radiotherapy
TME: tumor microenvironment

## Declarations

### Copyright

© The Author(s) 2025.
